# The *in Vitro* Biosynthesis of Chlorophyll *b* via Enzyme Catalysis

**DOI:** 10.1021/acscentsci.2c00997

**Published:** 2022-10-04

**Authors:** Linbin Niu, Yu Lan

**Affiliations:** † College of Chemistry and Institute of Green Catalysis, Zhengzhou University, Zhengzhou, Henan 450001, China; ‡ School of Chemistry and Chemical Engineering, Chongqing Key Laboratory of Theoretical and Computational Chemistry, Chongqing University, Chongqing 400030, China

Enzyme catalyzed chemical transformations provide sustainable strategies and enable synthetic approaches based on their powerful reactivity and potential for unique selectivity. However, the efficient biosynthesis of high-value biomolecules *in vitro* is still underexplored.^1^ As chlorophyll(ide) pigments are extensively used in the cosmetic, food, agricultural, and pharmaceutical industries,^2^ the custom-tuned synthesis of natural and unnatural chlorophyll(ide) derivatives *in vitro* is desirable. In this issue of *ACS Central Science,* Jennifer Bridwell-Rabb and co-workers employ four chlorophyll(ide) *a* oxygenase (CAO) homologues in cooperation with a non-native reductase to convert chlorophyllide *a* (Chlide *a*) into chlorophyllide *b* (Chlide *b*) *in vitro*.^1^

Chlorophylls are a class of naturally occurring pigments and serve key roles in photosynthetic organisms;^1,3^ however, their synthesis *in vitro* is complicated due to a lack of efficient protocols for *in vitro* reconstitution of an enzyme and demonstration of its activity in isolation.^3^ With the increasing demand for green and sustainable chemistry, chemists are pursuing those desirable systems that exclude noble metals, harsh conditions, toxic reagents, etc. In this context, tools and approaches from nature have provided inspiration to humans, especially the power of enzyme catalysis. Given the robust ability of chlorophylls to capture solar energy and transform it into chemical energy, the development of enzyme catalyzed efficient modification of high-value chlorophylls *in vitro* is of critical importance.

**Figure 1:**
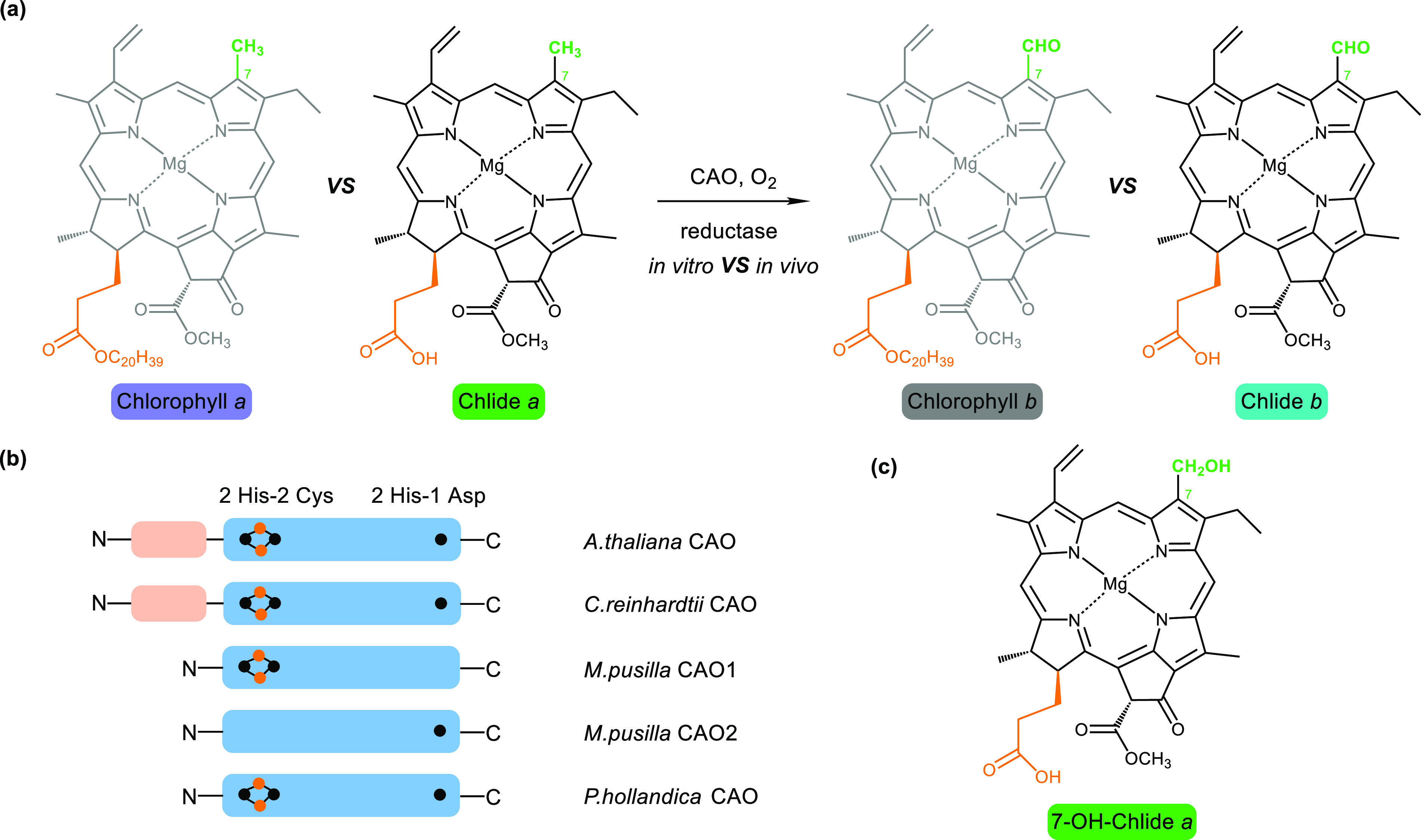
(a) CAO as a tool for formylation of chlorophyll scaffold. (b) The CAO homologues studied in ref (1) have different domain architectures. A Rieske [2Fe-2S] cluster and a mononuclear non-heme iron site are shown in blue. The N-terminal regulatory domains are shown in peach. (c) The structure of 7-OH-Chlide *a*.

As an annotated member of the large Rieske non-heme iron oxygenase protein family, CAO is capable of performing two sequential C(sp^3^)–H monooxygenation reactions on Childe *a* with O_2_ to afford Chlide *b*. This transformation requires the Rieske machinery in combination with a reductase system and proceeds through hydroxymethyl and dihydroxymethyl intermediates.^4,5^ Although CAO has shown the potential to catalyze late-stage oxidation reactions at different positions on the pigment scaffold,^1^ demonstrations of its catalytic activity *in vitro* are rare.^3^ The absence of protocols for the recombinant expression and purification of a CAO homologue and the lack of an annotated reductase hinder deep mechanistic exploration for chlorophyll *b* biosynthesis. To overcome these obstacles, the authors isolated and purified four CAO homologues that come from different kingdoms of life but possess conserved function in the conversion of Chlide *a* to Chlide *b* with the non-native Rieske reductases *in vitro* (Figure 1a,b). The results of the investigation into the promotion of the non-native Rieske reductases to the oxidation reaction showed that reactions conducted with VanB (the reductase partner of a Rieske oxygenase, vanillate *O*-demethylase) produced the highest amount of Chlide *b* when compared to DdmB/DdmA, spinach ferredoxin/ferredoxin reductase, *E. coli* flavodoxin/flavodoxin reductase and TsaB, owing to the ability of VanB to prohibit electrons from reacting with the Chlide *a* substrate, the protein, or an activated oxygen intermediate.^1^ The investigation of the substrate scope of Chlide *a* suggested the lack of a phytol chain, the presence of a central metal ion, and the electronics of the substrate may be important for achieving an efficient reaction with CAO. In contrast with the *in vitro* system, CAO could not convert chlorophyll *a* into chlorophyll *b* even in cell lysate.^6^ To deepen the fundamental understanding of chlorophyll biosynthesis and Rieske oxygenase chemistry, chlorophyllase was successfully applied to cleave chlorophyll into chlorophyllide, which facilitated subsequent detailed mechanistic investigations: (1) CAO is unable to abstract the hydrogen atom to serve as the initial step of the formylation reaction. (2) The MS experiments indicated 7-OH-Chlide *a* is a true intermediate of the oxygenation reaction (Figure 1c).^1^

Regarding the reductase system, we anticipate that exogenous light or electricity could serve as a potentially green, Earth-abundant, and sustainable reductant to replace the reductase, which might improve the yields,^7^ extend the substrate scopes, and generate new selectivity. Also, CAO has an obvious preference for the Chlide *a′* diastereomer,^8^ indicating that the stereochemistry in enzyme catalysis is also crucial. We believe theoretical calculations could make great contributions in the study of the stereoselectivity of biosynthetic enzymes, owing to the more credible and predictable transition states, intermediates, and reaction pathways.^9^ It could provide powerful support to understand the complicated chemistry of biomolecules and stimulate the exploration of more challenging and intricate biosynthetic transformations, especially enzyme-catalyzed transformations which proceed through radical intermediates with the potential to afford stereoenriched products.

This work by Jennifer Bridwell-Rabb and co-workers enriches the knowledge of chlorophyll biosynthesis, extends the known reactivity of the Rieske oxygenase class, and provides a framework for developing the CAO-VanB system as a tool to produce non-native chlorophyll pigments.^1^ The procedures established in this work for expressing, purifying, and reconstituting CAO homologues, and measuring the catalytic activity of CAO will have important implications for future structural and biochemical studies on enzyme catalysis. Determination of the key amino acids involved in the transformation of Chlide *a* into Chlide *b*, revealing clearer mechanisms and improving the yields and stereoselectivity, should be the focus of future research.
